# Hypoglycemic Mechanism of the Berberine Organic Acid Salt under the Synergistic Effect of Intestinal Flora and Oxidative Stress

**DOI:** 10.1155/2018/8930374

**Published:** 2018-12-19

**Authors:** Hong-Xin Cui, Ya-Nan Hu, Jing-Wan Li, Ke Yuan

**Affiliations:** ^1^College of Pharmacy, Henan University of Chinese Medicine, Zhengzhou 450046, China; ^2^Collaborative Innovation Center for Respiratory Disease Diagnosis and Treatment & Chinese Medicine, Development of Henan Province, Zhengzhou 450046, China; ^3^Forestry and Biotechnology College, Zhejiang Agriculture and Forestry University, Lin'an 311300, China; ^4^Jiyang College of Zhejiang Agriculture and Forestry University, Zhu'ji 311800, China

## Abstract

Both alterations to the intestinal microflora and chronic systemic inflammation predispose towards type 2 diabetes (T2D). Changes in the composition of the intestinal microflora are associated with glucose metabolism changes in rats with T2D. Here, we demonstrate that a berberine fumarate (BF) has a hypoglycemic effect by regulating the intestinal microflora and metabolism of diabetic rats. The T2D rats had disorders of glucose and lipid metabolism, an abnormal intestinal microflora, fewer butyrate-producing and probiotic-type bacteria, larger numbers of potentially pathogenic and sulfate-reducing bacteria, and tissue inflammation. Administration of berberine fumarate significantly ameliorated the metabolic disorder; increased the populations of Bacteroidetes, Clostridia, Lactobacillales, Prevotellaceae, and Alloprevotella; and reduced those of Bacteroidales, Lachnospiraceae, Rikenellaceae, and Desulfovibrio. In addition, it reduced inflammation, inhibiting the overexpression of TLR4 and p-JNK and increasing the expression of PI3K, GLUT2, and other proteins, which are closely related to oxidative stress, thereby promoting the metabolism of glucose.

## 1. Introduction

Type 2 diabetes (T2D) comprises a series of metabolic disorders caused by hypofunction of pancreas and insulin resistance. It is characterized by chronic hyperglycemia, which can result in many complications, such as heart disease, stroke, and chronic renal failure [1–3]. Not only is T2D associated with insulin resistance, but also it has been confirmed that changes in the intestinal microflora are involved in the low-grade chronic systemic inflammatory response that characterizes insulin resistance [4–6]. The microflora present in the human intestinal tract directly participate in digestion and absorption and influence energy metabolism in the body. Subtle changes in the species present and their proportions may lead to the development of various chronic metabolic diseases. In particular, accumulating evidence suggests that the intestinal microflora plays an important role in the pathogenesis of diabetes [7].

Therapeutic targeting of the microflora may have the potential to ameliorate insulin resistance and reduce the incidence of diabetic complications [8]. Conversely, disorders of the intestinal flora lead to inflammation and insulin resistance, exacerbating diabetes. Indeed, many studies have shown a correlation between changes in intestinal flora and markers of diabetes [9]. The intestinal microflora may influence the body mass, insulin sensitivity, bile acid metabolism, inflammation, and gastrointestinal hormone secretion by the host [10, 11]. Therefore, regulation of the intestinal microflora may be beneficial for glucose metabolism and ameliorate insulin resistance in the host. Release of lipopolysaccharide (LPS) by intestinal Gram-negative bacteria can initiate systemic inflammation, because LPS binds to and promotes the expression of CD14 and toll-like receptor 4 (TLR4), which activates an intracellular signaling pathway that leads to the secretion of proinflammatory cytokines. Oxidative stress is an imbalance in cellular redox reactions which plays a key role in the pathogenesis of metabolic disorders and drug-induced injury. Oxidative stress is the result of reactive oxygen species (ROS) overproduction or a decline in antioxidant defense mechanisms. Several diseases, including obesity, metabolic syndrome, diabetes mellitus, and others, are well-known to be associated with excessive ROS production. Thus, agents or signaling pathways that counteract excessive ROS and/or modulate oxidative stress represent an attractive strategy for treating T2D. The same study also showed that antibiotics have beneficial effects on the metabolic abnormalities in obese mice, ameliorating the impaired glucose tolerance, body mass gain, metabolic endotoxemia, inflammation, and oxidative stress [12].

Berberine is an isoquinoline alkaloid extracted from the rhizomes of *Ranunculaceae coptis* or *Cortex phellodendri*. A number of clinical studies have shown that berberine has a significant hypoglycemic effect in patients with T2D [13]. However, the oral bioavailability of berberine is low (0.68%), and about half of the dose is not absorbed by the intestines [14, 15]. Studies have shown that berberine organic acids can reduce blood glucose and have the good effects of berberine hydrochloride [13]. Fumaric acid, an intermediate product of the tricarboxylic acid (TCA) or Krebs cycle, has been widely used in food, sustainable chemistry, and biomedical applications, and fumarate is often used in the treatment of chronic inflammatory skin diseases and autoimmune diseases [16–18]. It has been shown that fumarate releases free radicals in the process of scavenging inflammation, thus protecting nerve and glial cells [19], while numerous clinical trials have demonstrated the safety and immunomodulatory effects of oral fumarate [20–22]. Therefore, the study's aim was to test if BF may have a beneficial effect on the composition of the intestinal flora in type 2 diabetic rats.

## 2. Materials and Methods

### 2.1. Materials and Animals

Streptozotocin (STZ) (purity > 98%) was purchased from Aladdin Bio-Reagent (Shanghai, China). ELISA kits were supplied by Jiancheng Biotech Sci. Inc., Nanjing, China.

Male Sprague-Dawley rats weighing 180–200 g were acclimatized to the experimental conditions of 20 ± 2°C, humidity 60 ± 5%, 12 h light/dark cycle, and *ad libitum* access to food and water. Rats and food (high-fat, high-sucrose diet containing 20% fat, 20% sucrose, and 2.5% cholesterol) were purchased from the Laboratory Animal Center of Zhejiang Academy of Medical Sciences (Zhejiang, China; certificate number SCXK 2014-0001). The study was approved by the animal ethics committee of Zhejiang Academy of Medical Sciences. All animal procedures were conducted in accordance with the Chinese animal care guidelines, which conform with internationally accepted guidelines for the use of experimental animals.

### 2.2. Experimental Design

Berberine fumarate (BF) was prepared by our laboratory to a purity of 98.8% [13]. T2D was induced by feeding rats with the high-fat, high-sucrose diet for 12 weeks, after which a single intraperitoneal injection of 50 mg/kg STZ dissolved in pH 4.2–4.8 citrate buffer solution (0.1 mol/L) was administered [23]. All rats were randomly divided into four groups (*n* = 8) as follows: NC (normal control) nondiabetic mice intragastric treated with distilled water, T2D rats intragastric treated with distilled water, 140 mg/kg·d metformin, or 500 mg/kg·d BF for 4 weeks.

After treatment was completed, blood was collected from a retro-orbital vein after 12–18 h of overnight fasting and centrifuged at 5120 × *g* for 10 min. Fasting blood glucose (FBG) and plasma levels of fasting insulin (FINS), triglycerides (TG), total cholesterol (TC), total superoxide dismutase (T-SOD), glycosylated serum protein (GSP), glutathione peroxidase (GSH-PX), glucagon-like peptide 1 (GLP-1), and LPS-binding protein (LBP) were measured using ELISA kits. The homeostasis model assessment for insulin resistance (HOMA-IR) was also calculated. At the end of the experiment, pancreas, liver, and ileum samples were promptly excised from animals anesthetized with 10% chloral hydrate solution. The samples were rinsed with normal saline and fixed in 10% neutral-buffered formalin for histopathological examination, following hematoxylin-eosin (H&E) staining. Fresh small intestinal contents were collected from the ileum in a sterile environment and stored at −80°C until use.

### 2.3. Analysis of the Composition of the Bacterial Microflora

Five samples from each group were used for analysis of the intestinal microbiota. Microbial genomic DNA was extracted from each fecal sample (0.1 g) using a Genomic DNA Isolation Kit (Sangon Biotech Co. Ltd., Shanghai, China). The construction of a high-throughput sequencing library and its sequencing using the Illumina MiSeq platform were completed by GENEWIZ (Suzhou, China). The concentration of DNA samples was measured using a Qubit 2.0 fluorometer (Invitrogen, Carlsbad, CA). The sequencing libraries were constructed using MetaVx™ Library construction kits (GENEWIZ Inc., South Plainfield, NJ, USA). Multiple variable regions of 16S rDNA (V3, V4) were amplified using DNA as a template. Sequence analysis was performed using VSEARCH (1.9.6) software. Sequences with ≥97% similarity were assigned to the same operational taxonomic unit (OTU). Taxonomic annotation was conducted using a ribosomal database project (RDP) classifier. Based on the results of the OTU analysis, alpha and beta diversity analyses were performed for all samples, to provide information about species richness, evenness, and differences in community structures.

### 2.4. Semiquantitative RT-PCR Analysis

Total RNA was isolated from ileum with TRIzol reagent (Sangon Biotech Co. Ltd., Shanghai, China). The expression levels of specific mRNAs were determined using semiquantitative RT-PCR analysis, normalizing to *β*-actin expression. One microgram of total RNA was reverse-transcribed using oligo dT and reverse transcriptase (Boya Co. Ltd., Shanghai, China). Then, cDNAs were amplified using oligonucleotide primers (Table 1) using a One-Step RT-PCR kit (Takara Co., Japan). The following PCR conditions were applied: denaturation at 95°C for 1 min, followed by 33 cycles (c-Jun n-terminal kinase (JNK) and phosphoinositol 3-kinase (PI3K): 95°C for 30 s, then 56°C for 1 min, and 72°C for 1 min) or 40 cycles (*β*-actin, TLR4, and facilitative glucose transporter 2 (GLUT2): 95°C for 30 s, then 60°C for 30 s), with a final extension at 72°C for 5 min. The PCR products were subjected to horizontal electrophoresis on 1.0% agarose gels, and images were captured using a Bio-Rad ChemiDoc imaging system (Hercules, CA, USA).

### 2.5. Western Blot Analysis

Ileal samples were homogenized in RIPA lysis buffer and centrifuged at 10001 × g (30 min, 4°C) to obtain cleared lysates. The supernatant protein concentrations were then measured using a BCA Protein Assay Kit (Aidlab Biotechnologies Co. Ltd., Beijing, China). For Western blot analysis, equal amounts of protein (50 *μ*g/lane) were electrophoresed on 12% polyacrylamide gels, after which they were electrotransferred onto polyvinylidene fluoride membranes (Millipore, Marlborough, MA, USA). Membranes were then incubated for 3 h in blocking buffer (1x tris-buffered saline containing 0.1% Tween-20, and 4% nonfat milk) at room temperature and then overnight in the same buffer containing primary antibodies against TLR4 (1: 1000), p-JNK (1: 1000), GLUT2 (1 : 1000), PI3K (1 : 500), or *β*-actin (1: 1500) (Boster Biological Technology Ltd., Wuhan, China). For antibodies targeting phosphorylated proteins, bovine serum albumin (BSA) was used instead of nonfat milk. Membranes were then washed three times for 5 min and incubated for 2 h at 4°C with HRP-conjugated secondary antibodies (anti-rabbit or anti-rat) (Boster Biological Technology Ltd., Wuhan, China). Proteins were detected using an enhanced chemiluminescence detection system (Amersham Pharmacia, Piscataway, NJ, USA) [24].

### 2.6. Statistical Analysis

All data are expressed as mean ± standard deviation (SD) and were analyzed using SPSS statistical software (SPSS 19.0, SPSS Inc., Chicago, IL). One-way analysis of variance (ANOVA) with Duncan's test was used for intergroup comparison. *P* < 0.05 was considered to represent statistical significance.

## 3. Results and Discussion

### 3.1. The Effect of BF on Biochemical Indices in the Plasma of T2D Rats

Many studies have shown that berberine is an effective antihyperglycemic agent and has positive effects on diabetic complications, such as hypertension, hyperlipidemia, cardiovascular and cerebrovascular diseases, and peripheral nerve lesions [13, 25]. However, we hypothesized that the combination of berberine and fumaric acid could be safer and more effective [13].

**Figure 1:**
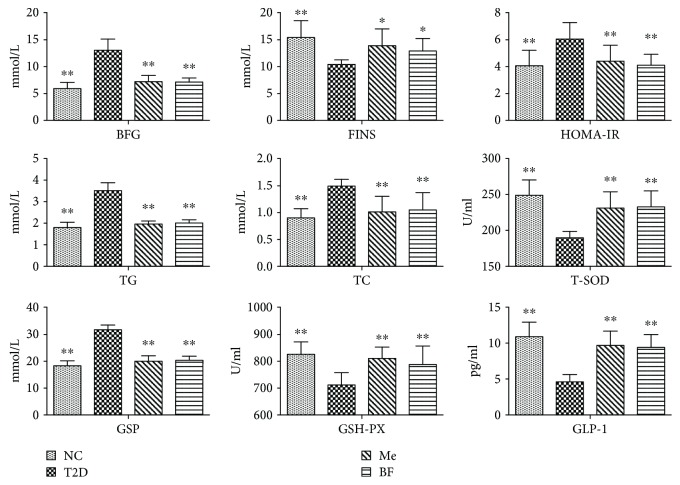
The effect of BF on the plasma index of T2D rats. The data were expressed as mean ± SD (*n* = 8), ^∗^ *P* < 0.05 and ^∗∗^ *P* < 0.01 vs T2D group.

As shown in Figure 1, BF treatment significantly increased plasma FINS, T-SOD, GSH-PX, and GLP-1 and reduced FBG, HOMA-IR, and plasma TG, TC, and GSP (all *P* < 0.05) in diabetic rats. Berberine can inhibit gluconeogenesis by regulating the function of *β* cells and can also promote glycolysis, thereby lowering blood glucose and lipid levels and ameliorating insulin resistance [26]. The effect of berberine to reduce blood lipid is the result of an improvement in antioxidant capacity, the promotion of lipid metabolism, and the inhibition of preadipocyte differentiation [13, 25]. Excessive oxygen-free radicals can trigger insulin resistance by modulating signal transduction [27], and insulin resistance may aggravate oxidative stress [28].

It has also been shown that berberine can ameliorate abnormalities in plasma gastrointestinal hormone levels, such as those of glucagon-like peptide- (GLP-) 1 and 2, insulin stimulating polypeptide [29], and pancreatic polypeptide. GLP-1 is mainly secreted by L cells distributed throughout the ileum. Berberine can increase the proliferation of L cells, increase glucagon and prohormone invertase synthesis, and enhance GLP-1 secretion in diabetic rats [30]. Therefore, BF may regulate glucose and lipid metabolism by promoting the synthesis and secretion of GLP-1 in the intestinal tract.

Many studies have shown that the human intestinal microflora can convert insoluble nutrients, such as proteins and carbohydrates, into soluble substances, to facilitate their absorption. It can also transform difficult-to-absorb polysaccharides into monosaccharides, metabolize glucose to form lactic acid, and participate in the metabolism of cholesterol, thereby having an important effect on the nutrients present in and absorbed from the intestine [31]. Microbiological studies have shown that there are significant differences in the type and number of intestinal bacteria in the intestines of diabetic patients and in those of healthy people [32, 33]. One study has also shown that the secretion of GLP-1 and other hormones is lower in high-fat diet-fed mice, while feeding additional dietary fiber or probiotics can significantly increase the secretion of the same hormones [34, 35], suggesting that the secretion of gastrointestinal hormones is influenced by changes in the intestinal microflora. Also, there was no significant difference between the BF group and the Me group.

### 3.2. Effect of BF on the Intestinal Microflora of Rats with T2D

The normal intestinal flora forms a natural barrier on the surface of the intestinal mucosa and participates in normal digestion and absorption in humans, but it also regulates immune function and prevents the invasion of pathogenic bacteria and opportunistic pathogens [36]. Thus, the intestinal microflora directly participates in nutrient digestion and absorption, energy supply, fat metabolism, immune regulation, and disease resistance [37, 38]. In addition, disorders in the composition of the microbiota may also be involved in the development of chronic metabolic diseases, such as obesity [39, 40] and diabetes [41, 42]. Previous findings have suggested that the beneficial effects of metformin (Me) on glucose metabolism may be in part microbially mediated [43, 44]. Berberine has been used clinically for many years to treat bacterial infections in the intestines, but in addition, after 4 weeks of berberine administration to high-fat diet-fed mice, their body mass, visceral fat content, blood glucose, and plasma lipid content had been reduced significantly. However, these effects were also associated with significant reductions in the proportions of Firmicutes and Bacteroidetes in the feces of the mice [45].

**Figure 2:**
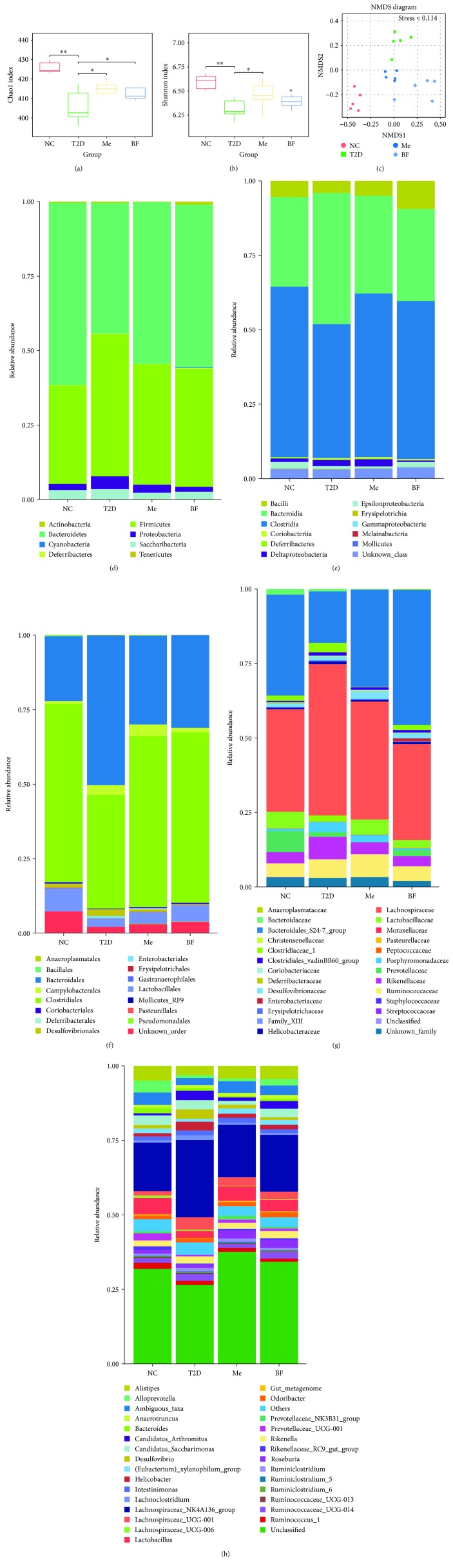
The effect of BF on the structure of intestinal flora of T2D rats. (a, b) Analysis of alpha diversity; (c) analysis of beta diversity; (d–h) the relative abundances of main species under different levels (phylum, class, order, family, and genus). *n* = 5, ^∗^ *P* < 0.05 and ^∗∗^ *P* < 0.01.

Here, alpha diversity analysis showed that the bacterial species richness and diversity in the intestines of the T2D group were significantly lower than those of the control rats (*P* < 0.05), but that these were largely normalized by treatment with metformin and BF (Figure 2). Nonmetric multidimensional scaling (NMDS) plots of these data are shown in Figure 2(c). The stress for these was <0.114, indicating that NMDS accurately reflects the degree of difference between samples, which is indicated by the distance between each point. This technique demonstrates a clear difference between the T2D group and the other groups. Previous studies have shown that berberine administration can reduce the diversity of the intestinal microflora in rats and selectively increase the abundance of bacteria producing short-chain fatty acids, such as Blautia and Allobaculum [46].

As shown in Figure 2, representatives of eight main phyla were detected in the small intestinal contents of each group: Firmicutes, Bacteroidetes, Saccharibacteria, Proteobacteria, Actinobacteria, Deferribacteres, Tenericutes, and Cyanobacteria. Members of the Firmicutes and Bacteroidetes were dominant in all the groups. However, the relative abundance of Bacteroidetes in the T2D group (44.07%) was lower than in the NC group (61.46%), but this difference was largely abolished after treatment (Me: 54.54%; BF: 54.77%). An imbalance in the proportions of Firmicutes and Bacteroidetes in the intestinal microflora is associated with many diseases. The ratios of Firmicutes to Bacteroidetes (F/B) in the NC, T2D, Me, and BF groups were 0.54, 1.09, 0.74, and 0.73, respectively (Figure 2(d)). Previous studies have shown that Bacteroidetes are more abundant in patients with diabetes, such that the F/B ratio is lower [47]. Here, we show that the F/B ratio in the rat intestines was positively correlated with their host's blood glucose concentrations (*P* < 0.01) (Table 2), which is consistent with the findings of previous studies [48]. However, no significant correlation was found between F/B ratio and blood lipids, oxidative stress, and GLP-1.

The Clostridia, Bacteroidia, and Bacilli were the dominant bacterial classes identified. Compared with the NC group (55.56%, 29.23%), the relative abundance of Clostridia (44.24%) was lower and that of the Bacteroidia (42.77%) was higher, in the T2D group, but these differences were largely abolished by treatment (Me: 53.34%, 31.89%; BF: 51.50%, 30.01%) (Figure 2(e)). In addition, the relative abundance of Clostridia in the rat intestines was negatively correlated with their host's blood glucose [49, 50], as shown in Table 2 (*P* < 0.05). Previous studies have shown that Clostridiales and Lactobacillales can ferment saccharides to form butyric acid or conjugate oleic acid, which can contribute to cell differentiation [51]. The present study shows that the Clostridiales and Bacteroidales were the dominant orders in the intestines of each group of rats. In addition, compared with the NC group (56.34%, 7.33%, and 20.53%), the relative abundances of Clostridiales (35.91%) and Lactobacillales (2.46%) were lower and that of Bacteroidales (47.31%) was higher, in the T2D group, and these differences were largely abolished by treatment (Me: 54.06%, 3.75%, and 28.14%; BF: 53.89%, 5.05%, and 29.26%) (Figure 2(f)). Moreover, there was no significant difference between the BF group and the Me group.

Previous studies have shown that lactic acid produced by bacteria can be used by other bacteria to produce butyric acid in the intestine, thereby promoting intestinal synthesis of mucin, which protects the intestinal mucosa [52]. Berberine has been shown to reduce the abundance of butyric acid-producing bacteria in the Clostridium coccoides and Clostridium leptum subgroups, and this is significant because butyric acid bacteria can degrade plant polysaccharides to provide additional energy for the host. Roseburia are butyric acid-producing bacteria, while Prevotella can cause the degradation of mucin. Previous animal experiments have shown that the presence of large numbers of Lactobacilli and Bifidobacteria are associated with diabetes resistance, while large numbers of Bacteroides and IV Clostridium can promote the development of diabetes [53]. Bacteroides and IV Clostridium can use glucose and lactic acid to synthesize short-chain fatty acids which cannot be used for mucin synthesis [54], but instead increase the permeability of the intestinal mucosa and promote inflammation [52]. These findings regarding the effects of specific bacterial groups have laid the foundation for the targeting of the intestinal microflora in T2D therapy.

In Figure 2(g), the Lachnospiraceae and Bacteroidales S24-7 are shown to be the dominant bacterial types in each rat group. Compared with the NC group (30.25%, 3.34%, 29.84%, and 6.34%), the relative abundances of Lachnospiraceae (44.58%) and Rikenellaceae (3.55%) were higher and those of Bacteroidales S24-7 (15.21%) and Prevotellaceae (1.47%) were lower, in the T2D group. In addition, there were also different distributions of families and genera within a phylum. For example, with regard to Bacteroidetes, compared with the NC group (0.58%), the relative abundance of Bacteroidaceae (1.77%) was higher, but those of S24-7 and Rikenellaceae were lower in the T2D group. *Prevotella copri* and *Bacteroides vulgatus* have previously been identified as the main species responsible for the association between the biosynthesis of branched-chain amino acids (BCAAs) and insulin resistance, and in mice we have demonstrated that *P. copri* can induce insulin resistance, aggravate glucose intolerance, and augment circulating levels of BCAAs [8].

Figure 2(h) shows that the abundance of the Desulfovibrio genus (2.69%) of Proteobacteria was higher and that of Alloprevotella (0.92%) of Bacteria was lower in the T2D group than in controls (0.85%, 3.34%). Desulfovibrio species are sulfate-reducing bacteria that reduce sulfate to sulfide in the intestines. Sulfides have toxic effects on intestinal epithelial cells [55], inducing abnormal proliferation and metabolism of epithelial cells, which impairs intestinal barrier function [56, 57]. In addition, the abundance of Epsilon-proteobacteria is positively correlated with FBG (*P* < 0.05) (Table 2).

Previous studies have shown that hyperglycemia can result from a decrease in the proportion of anaerobes, especially Bacteroides [9]. Therefore, regulation of the intestinal microflora may be beneficial to the glucose metabolism and insulin sensitivity of the host. Furthermore, the study has shown that abundance of Lactobacilli positively correlates with FBG and glycosylated hemoglobin levels and that of Clostridia negatively correlates with FBG, glycosylated hemoglobin, and insulin levels [58]. As shown in Table 2, the abundance of Clostridia and Bacillales are negatively correlated with FBG (*P* < 0.05), while there is no clear correlation between the abundance of Lactobacilli and FBG (*P* > 0.05), perhaps because of the large number of changes in the composition of intestinal microflora. A considerable number of bacterial types used in probiotics, such as Bifidobacteria and Lactobacilli, exist in the intestinal tract of healthy people. However, the number of such bacteria in diabetic patients is significantly lower than that in healthy individuals, and therefore their numbers may be negatively correlated with FBG [59–61]. Studies have shown that high-fat diet leads to intestinal flora structure disorder by inducing oxidative stress, and intestinal flora can significantly regulate lipid metabolism [10]. However, no significant correlation was found between oxidative stress and lipid metabolism and bacteria.

### 3.3. The Effect of BF Administration on the TLR4/JNK/PI3K Signaling Pathway

**Figure 3:**
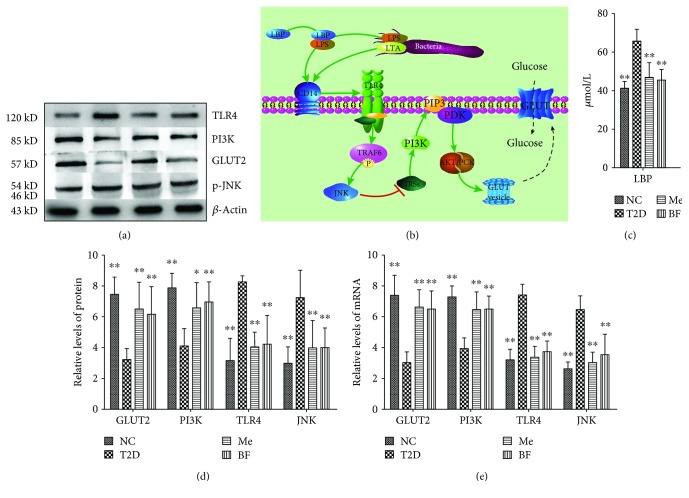
The results of Western blot and RT-PCR. (b) Signal pathways, “↑,” activation and “T,” inhibition. The data were expressed as mean ± SD (*n* = 8), ^∗^ *P* < 0.05 and ^∗∗^ *P* < 0.01 vs T2D group.

The effect of BF on insulin resistance in rats with T2D was at least partially mediated through effects on the intestinal microflora. In particular, changes in the intestinal flora can reduce the level of plasma lipopolysaccharide-binding protein (LBP) being produced and thereby help to reduce systemic inflammation (Figure 3).

The c-Jun n-terminal kinase (JNK) plays a vital role in the metabolic changes and inflammation induced by a high-fat diet, which are involved in the pathogenesis of T2D [62–64]. Under diabetic conditions, the JNK pathway is activated in various tissues and has deleterious effects on both insulin sensitivity and pancreatic *β*-cell function. Activation of the JNK pathway interferes with insulin action and reduces insulin biosynthesis, and its inhibition in diabetic rats ameliorates insulin resistance and *β*-cell function, leading to an improvement in glucose tolerance. Thus, the JNK pathway is likely to play a central role in the progression of insulin resistance and *β*-cell dysfunction and could represent a potential therapeutic target for T2D [64].

The PI3K pathway is the main signal transduction pathway mediating insulin action in the liver, and inhibition of PI3K signaling leads to insulin resistance and potentially therefore obesity, fatty liver, T2D, and metabolic syndrome [65, 66]. In particular, downregulation of insulin receptor substrate- (IRS-) 2 can impair the effective transmission of the downstream PI3K signal and lead to insulin resistance [67–69]. GLUT2, the main glucose transporter in rodents and human hepatocytes, mediates the bidirectional transport of glucose, and therefore, its abnormal expression can lead to disorders of glucose metabolism.

As shown in Figure 3, LBP, TLR4, and JNK expressions were significantly higher, and GLUT2 and PI3K expressions were significantly lower, in T2D rats than in control rats, and these differences were largely abolished by BF treatment (*P* < 0.05). This suggests that BF may alleviate the inflammatory response and ameliorate insulin resistance through effects on the JNK and PI3K signaling pathways that are likely to improve glucose metabolism (Figure 3(b)).

In addition, oxidative stress is one of the key factors for the development of insulin resistance. A high-fat diet can induce more reactive oxygen species in the body, which can activate multiple intracellular signaling pathways, cause disorder of lipid metabolism, and produce insulin resistance [70]. In a high-glucose environment, cell inflammation is enhanced, and inflammatory cells can produce a large number of oxides, making oxidative stress possible.

### 3.4. The Protective Effect of BF on the Tissues of Diabetic Rats

Berberine can ameliorate ileal and systemic inflammation by inhibiting the activation of the TLR4 pathway and reducing the intestinal damage caused by LPS [71]. A microscopic examination of the liver of NC rats demonstrated a normal central vein and a narrow surrounding sinusoidal radiation, without any significant congestion of the liver sinuses or cell swelling. In contrast, the liver of STZ-induced diabetic rats showed obvious pathology, in the form of nonradiating sinusoids, scattered necrotic cells showing pyknosis, and the formation of microvesicles in the cytoplasm of hepatocytes. However, the histopathology in the liver of treated rats was variable, but less marked.

**Figure 4:**
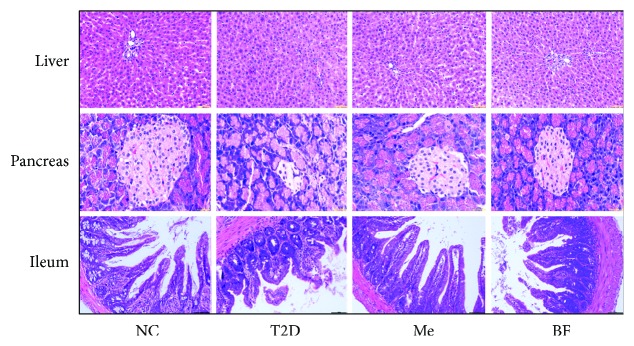
The protective effect of BF on liver, pancreas, and ileum of T2D rats (hematoxylin-eosin stain, ×200).

The pancreatic islets of rats in the NC group appeared normal, whereas those of diabetic rats showed severe pathology, including necrosis, smaller size, and fewer cells. The pancreatic islets of rats in the BF and Me groups also showed injuries, but there was a significant improvement compared with the T2D group (Figure 4).

The ileal villi of the NC group were arranged neatly and compactly, with no endothelial discontinuities. In contrast, the ileal villi of diabetic rats were fewer in number, blunted, and lacked structural integrity, while mucosal inflammation was also apparent. However, in rats that had been treated for 6 weeks with BF and Me, the ileal villi were neatly arranged and more intact and numerous than those of the T2D group (Figure 4). When the organism is infected by gram-negative bacteria, lipopolysaccharide can activate signaling pathways, resulting in a large number of proinflammatory cytokines released. Berberine can reduce the release of lipopolysaccharide and ameliorate inflammation by reducing the level of LBP, thus alleviating intestinal injury and improving insulin resistance [72].

## Figures and Tables

**Table 1:** Sequence of primers used for the RT-PCR assays.

Genes	Primer sequences	
	Forward primer (5′→3′)	Reverse primer (5′→3′)
TLR4	ATCATCCAGGAAGGCTTCCA	GCTGCCTCAGCAAGGACTTCT
JNK	TGACGCCTTATGTGGTGACT	TGATGTATGGGTGCTGGAGA
GLUT2	CCTGCTTGGTCTATCTGCTGTG	CCTTGCTTTGGCTTCCCC
PI3K	CATCACTTCCTCCTGCTCTAT	CAGTTGTTGGCAATCTTCTTC
*β*-Actin	GCCATGTACGTAGCCATCCA	GAACCGCTCATTGCCGATAG

**Table 2:** Analysis of the correlation between abundance of intestinal flora and fasting blood glucose (FBG).

		F/B	Clostridia	Epsilon-proteobacteria	Bacillales	Lactobacilli
FBG	P	0^∗∗^	0.012^∗^	0.001^∗^	0.006^∗^	0.0541
	r	0.763	−0.552	0.683	−0.596	0.501

## Data Availability

All the figures and tables used to support the findings of this study are included within the article.

## Abbreviations

T2D: Type 2 diabetes
STZ: Streptozotocin
BOAS: Berberine organic acid salt
BF: Berberine fumarate
NC: Normal control
Me: Metformin
FBG: Fasting blood glucose
FINS: Fasting insulin
TG: Triglycerides
TC: Total cholesterol
T-SOD: Total superoxide dismutase
GSP: Glycosylated serum protein
GSH-PX: Glutathione peroxidase
HOMA-IR: The homeostasis model assessment for insulin resistance
GLP-1: Glucagon-like peptide 1
LPS: Lipopolysaccharide
LBP: LPS-binding protein
JNK: c-Jun n-terminal kinase
PI3K: Phosphoinositol 3-kinase
GLUT2: Glucose transporter 2
TLR4: Toll-like receptor 4.

## Additional Points

*Chemical Compounds.* Chemical compounds mentioned in this study are berberine hydrochloride (PubChem CID: 12456), fumaric acid (PubChem CID: 444972), streptozotocin (PubChem CID: 29327), sodium chloride (PubChem CID: 5234), water (PubChem CID: 962), citric acid (PubChem CID: 311), butyric acid (PubChem CID: 264), metformin (PubChem CID: 4091), alpha-D-glucose (PubChem CID: 79025), and triglyceride (PubChem CID: 10851).

## Supplementary Materials

Supplementary Materials The corresponding experimental data associated with this article can be seen from the supplementary material files.
